# First COVID-19 Vaccines Receiving the US FDA and EMA Emergency Use Authorization

**DOI:** 10.15190/d.2021.1

**Published:** 2021-03-05

**Authors:** Andra Fortner, David Schumacher

**Affiliations:** ^1^Albert-Ludwigs-Universität Freiburg, Germany; ^1^Department of Anesthesiology, University Hospital RWTH Aachen, Germany

**Keywords:** COVID-19, vaccine, mRNA vaccine, US Food and Drug Administration, European Medicines Agency.

## Abstract

On December 31, 2019, the Wuhan Municipal Health Commission reported an increase in the incidence of pneumonia from an unknown cause. Shortly after, SARS-CoV-2 was identified as the responsible coronavirus for the heavy progress of the disease, which can manifest itself distinctively in different individuals. Coronavirus Disease 2019 (COVID-19) triggered a pandemic because of its high contagiousness before COVID-19 associated symptoms actually appear. In response to the rapid and continuous spread of the virus around the globe governments have mobilized their forces to restrict contact and thus avoid further infection and invested significant resources in treatment and prevention strategies to tackle COVID-19. As a result, US FDA and EMA have granted emergency use authorization for two mRNA-based vaccines, namely the vaccines developed by BioNTech/Pfizer and Moderna, for use in the USA and Europe. Due to the existing critical situation, the stages of vaccine development and testing have probably never been gone through so fast as at present. Here, we are briefly commenting on these two vaccines with their benefits, advantages and limitations.

## 1. Introduction

Public interest in mRNA vaccines has arisen greatly since the global pandemic of COVID-19. As of February 20, 2021, more than 2.435.000 people have died from or with COVID-19 all over the world and more than 109.900.000 have been infected^1^.

Although there are more than 50 vaccines in clinical trials and more than 240 in pre-clinical development^2-5^, only two vaccines have been authorized for emergency use by the U.S. Food and Drug Administration (FDA) and European Medicines Agency (EMA) until January 2021, these vaccines, therefore, being in the center of attention to contribute to the halt of the uncontrollable spread of the pandemic (Table 1).

**Table 1:** Promising vaccines, in use, authorized and in clinical trials Modified with permission from^2-5^.

VACCINE	COMPANY/ DEVELOPER	TYPE OF VACCINE	STATUS
MRNA 1273	Moderna/National Institute of Allergy and Infectious Diseases (NIAID)/Biomedical Advanced Research and Development Authority (BARDA)/ Lonza/ Catalent/Rovi/ Medidata/ BIOQUAL	mRNA-based vaccine	In use in the US, Canada, the EU, the UK, Switzerland, Israel
BNT162	BioNTech/ Pfizer/ Fosun Pharma/ Rentschler Biopharma	mRNA-based vaccine	In use by the European Commission, in the US, UK, Canada, Argentina, Mexico, Saudi Arabia, Bahrain
AZD 1222 (FORMERLY CHADOX1)	University of Oxford, Oxford Biomedica, Vaccines Manufacturing and Innovation Centre, Pall Life Sciences, Cobra Biologics, HalixBV, Advent s.r.l., Merck KGaA, the Serum Institute, Vaccitech, Catalent, CSL and AstraZeneca/IQVIA	Non-Replicating Viral Vector	One of the largest vaccination programs; in use in the UK, EU, Argentina, Brazil, Dominican Republic, El Salvador, India, Mexico, Marocco, Pakistan
Janssen’s COVID-19 vaccine (AD26.COV2-S)	Janssen Pharmaceutical Companies/Johnson & Johnson/Beth Israel Deaconess Medical Center/ Emergent Biosolutions/ Catalent/ Biological E/ Grand River Aseptic Manufacturing (GRAM)	Non-Replicating Viral Vector (alone or with modified vaccinia Ankara (MVA) boost)	Phase III clinical trial Approved for emergency use in the USA on February 28, 2021.
GAM-COVID-VAC (SPUTNIK V)	Gamaleya Research Institute	Non-Replicating Adenoviral Vector	In use in Algeria, Argentina, Bolivia, Hungary, Palestine, Paraguay, Serbia, Turkmenistan, UAE, Venezuela; “registered” in Belarus and Russia
AD5-NCOV	CanSino Biologics/ Beijing Institute of Biotechnology/ Petrovax	Non-Replicating Viral Vector (adenovirus type 5 vector)	In use for “the military” in China
To be defined	Wuhan Institute of Biological Products/ Sinopharm	Inactivated Virus	Authorized for emergency use in China and the UAE
CORONAVAC (FORMERLY PICOVACC)	Sinovac/ Instituto Butantan/ Bio Farma	Inactivated Virus	Authorized for emergency use in Brazil, China, Indonesia
BBIBP-CORV	Beijing Institute of Biological Products/ Sinopharm	Inactivated Virus	In use in Bahrain, China, Pakistan, the UAE
INO-4800	Inovio Pharmaceuticals/ Beijing Advaccine Biotechnology/ VGXI Inc./ Richter-Helm Biologics/ Ology Bioservices/ International Vaccine Institute/ Seoul National University Hospital/ Thermo Fisher Scientific/ Kaneka Eurogenetec	DNA plasmid vaccine with electroporation	Phase II/III clinical trial
NVX-COV2373	Novavax/ Emergent Biosolutions/ Praha vaccines/ Biofabri/ Fujifilm Diosynth Biotechnologies/ FDB/ Serum Institute of India/ SK Bioscience/ Takeda Pharmaceutical Company Limited/ AGC Biologics/ Polypeptide Group/ Endo	Protein subunit (full length recombinant SARS-CoV-2 glycoprotein nanoparticle vaccine adjuvanted with matrix M)	Phase III clinical trial

Both vaccines are based on a new mRNA technology as their active agent, and therefore, take a new approach to vaccination. Unlike traditional forms of vaccinations that use inactivated, live-attenuated or a sole antigen of a virus, new vaccination methods are currently being researched. Apart from mRNA-based vaccines, those methods include DNA-based vaccines, viral vectors or the injection of antigen-presenting cells^3^.

**Figure 1:**
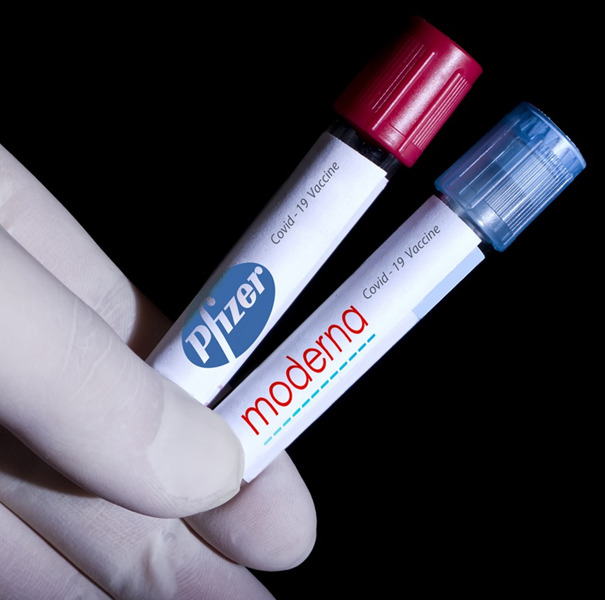
First two vaccines receiving emergency use authorization in Europe and the USA Photo provided by dreamstime.com

While the first approved mRNA-based vaccine in Europe and USA named BNT162b2, which is also known under the name Comirnaty, has been developed by the German company BioNTech in collaboration with the USA company Pfizer, the other vaccine, mRNA-1273 COVID-19, was developed in Cambridge, MA, USA^4-9^ (Figure 1, Table 1).

## 2. Vaccines approved for emergency use by US FDA and EMA

The first vaccine receiving emergency authorization by the FDA on December 11, 2020^6^, and by the EMA on December 21, 2020^7^, is the mRNA-based vaccine Comirnaty from Pfizer and BioNTech. Shortly after, the FDA and the EMA granted Moderna’s vaccine access on the market on December 18, 2020^8^, and January 06, 2020 respectively^9^ (Figure 1).

Both vaccines contain a mRNA single-strand coding for the spike protein^10,11^ of the SARS-CoV-2. This type of protein can be found on the virus’ surface and it is known to allow the coronavirus to enter the host cells^12,13^. The mRNA itself is made up of a 5’ cap, UTR sequences before and after the actual coding sequence and a poly(A) tail at the 3’ end of the single strand. These structures provide the molecule stability in the cytoplasm and promote the process of translation^14^. Furthermore, the two vaccines are nucleoside-modified, meaning that some single RNA nucleotides have been exchanged in order to create codons coding for the same amino acid. These new codons, however, are more efficiently translated because their corresponding tRNA molecule can be found in greater amounts inside the cell.

Another important component of the vaccines are lipid nanoparticles (LNPs) enclosing the mRNA. Their function refers to the in vivo delivery of the mRNA: on the one hand, LNPs prevent the nucleic acid from being degraded by RNAses in the extracellular space and on the other hand, they aid the uptake of the mRNA into cells at the injection site^15^. The two FDA and EMA authorized vaccines are very similar but structurally differ in the type of lipid used for the lipid nanoparticles^16^.

Once the mRNA has been released in the cytoplasm of the target cell, it will pass through the process of translation, creating the SARS-CoV-2 spike protein’s peptide chain that will be properly folded in further steps. The mRNA strand itself is broken down after translation. Body cells display antigens on special cell receptors (MHC) on the cell surface, where they can be recognized by human immune cells. This generates a humoral and cell-mediated immune response by activating different types of antigen-specific lymphocytes (B and T cells). B-cells produce high amounts of antibodies that can bind the epitopes of the vaccinated antigen in order to neutralize it, whereas cytotoxic T-cells specifically kill body cells presenting the antigen on their MHC receptors. The remaining antibodies and immune cells continue to circulate through the body. They are the basis for immunity against SARS-CoV-2^10,11^.

In the case of a secondary infection, the virus can quickly be recognized and is neutralized at the epitopes of its spike protein by antibodies^17^. At the same time, cytotoxic T-cells specifically spot infected body cells and kill them to prevent the spreading of the SARS-CoV-2 infection inside the body. Hence, the virus is destroyed and the onset of COVID-19 disease symptoms can be prevented^18^.

Both vaccines - BNT162b2 and mRNA-1273 - are administered two times. The second injection of the vaccine should mimic a secondary infection by inducing new production of the antigen and serves as a booster to obtain a greater immunity against SARS-CoV-2^19,20^. According to the vaccination schedule the second dose of BNT162b2 is given after 21 days, whereas mRNA-1273 is readministered after 28 days^21,22^. The two vaccines also differ as BNT162b2 has to be diluted prior to use^21^.

Several other vaccines are now evaluated/ approved by the FDA and EMA, including the Janssen’s COVID-19 vaccine (AD26.COV2-S) developed by the Janssen Pharmaceutical Companies (Johnson and Johnson) and their partners (such as Beth Israel Deaconess Medical Center, Boston, USA), approved for emergency use by FDA on February 28, 2021 and AZD 1222 developed by the University of Oxford with AstraZeneca and their partners (see Table 1). On January 29, 2021, EMA has recommended granting a conditional authorization for AZD 1222 vaccine use in Europe.

There are many advantages, benefits, but also limitations for the mRNA vaccines, which are discussed in the next section.

## 3. Advantages and Limitations

Similar to any other studied immunogenic substances and approaches, there are aspects that play in favour of or against the usage of mRNA vaccines.

### 3.1 Efficacy

Until now, both vaccines have been shown to be highly effective^19-22^. Clinical trials of BioNTech/Pfizer and Moderna suggest that both vaccines prevent COVID-19 more or less equally well (95%, respectively 94,1% efficacy^19^). Both trials were conducted randomized, placebo-controlled and observer-blinded. A total of 43,448 volunteers participated in the clinical trial of BioNTech/Pfizer, whereas Moderna’s clinical trial counted a smaller number of individuals (30,420). In each study, the number of participants has been divided in half; one group being injected with the mRNA-based vaccine, while the other group was given a placebo. All test subjects have been tested for SARS-CoV-2 infection after receiving the second dose of vaccine, respectively placebo: seven days after the second administration in BioNTech/Pfizer’s study and 14 days after the second administration in Moderna’s study^19^. 162 infected people have been confirmed in the placebo group and only 8 people in the BNT162b2 group. These numbers have been calculated to an efficacy of 95%. In the other trial there have been 185 infections in the placebo group and only 11 in the group receiving Moderna’s mRNA vaccine, resulting in an efficacy of 94,1%^23,24^.

Both test results reveal that the vaccines’ efficacy is not influenced by age, gender, ethnicity or pre-existing illnesses of individuals^21,22^.

In comparison to the conventional way of injecting the antigen only, mRNA vaccines may provide a stronger type of immunity because not only antibodies are built in a humoral immune response, but also specific killer cells come into action^23,24^.

However, many new variants of the SARS-CoV-2 have evolved recently. This presents a challenge due to the possibility of increased contagiousness of new SARS-CoV-2 variants that can develop because of random mutations in the virus’ genome. Some examples are the B.1.1.7 variant, first observed in the UK; variant B.1.351, discovered in South Africa or variant B.1.1.28 P.1, first found in Brazil^25^. Questions are raised on whether the two mRNA-based vaccines or the other vaccines are able to generate immunity efficiently against mutated SARS-CoV-2 virus, because the spike protein’s structure of the mutated virus might too greatly differ from the spike protein encoded by the vaccines. Further research into this area is required.

### 3.2 Safety

Although it is true that many of the exact biological processes of the respective substances and immunity are not fully understood, it is important to note that research into this type of vaccine has already started years ago and the vaccines have continuously been improved since then^15^. Additionally, clinical trials have been and are still being conducted in order to determine whether vaccines can meet the standards set by the responsible institutions^26^.

Since the vaccine is based on mRNA, the vaccine itself is not infectious and concerns on the genetic piece being inserted into our DNA-genome are unfounded^14^. In addition, mRNA has an only short halflife in the cytoplasm as it is degraded by enzymes after translation^14^. Despite this, and especially as all organisms respond differently to the same substance, adverse effects can still occur because of unintended immune reactions to the mRNA strand^27^.

Regarding the reactogenicity of the two FDA and EMA authorized vaccines, adverse effects in the clinical trials were usually mild or moderate in intensity and resolved after vaccine is administered in few days^21,22^, whereby these effects generally occured more often in younger people and after the second dose of vaccine^21,22^. Side effects include pain at the injection site, fatigue, headache, muscle pain, joint pain and chills (see Table 2) and indicate that an immune response is generated^19-22^.

**Table 2:** Pfizer and Moderna vaccines Information adapted with permission from^4,19,21,22^

Name	COMIRNATY concentrate for dispersion for injection COVID-19 mRNA Vaccine (nucleoside modified)	COVID-19 Vaccine Moderna dispersion for injection COVID-19 mRNA Vaccine (nucleoside modified)
Age indication	16 years old and older	18 years old and older
Vaccination schedule	2nd dose after 21 days	2nd dose after 28 days
Dosage	30 micrograms of COVID-19 mRNA Vaccine (embedded in lipid nanoparticles)	100 micrograms of messenger RNA (mRNA) (embedded in SM-102 lipid nanoparticles)
Administration	intramuscularly	intramuscularly
Efficacy	~ 95%	~ 94,1%
Undesired/adverse effects in the clinical trial(s)	pain at the injection site pain (> 80%), fatigue (> 60%), headache (> 50%), myalgia and chills (> 30%), arthralgia (> 20%), pyrexia and injection site swelling (> 10%).	pain at the injection site (92%), fatigue (70%), headache (64.7%), myalgia (61.5%), arthralgia (46.4%), chills (45.4%), nausea/vomiting (23%), axillary swelling/tenderness (19.8%), fever (15.5%), injection site swelling (14.7%), redness (10%).
Storage	Unopened vial: 6 months at -90 °C to -60 °C, up to 5 days at 2 °C to 8 °C, up to 2 hours up to 30 °C, protected from light. After dilution: 6 hours at 2 ºC to 30 ºC.	Unopened vial: 7 months at -25ºC to -15ºC, 30 days at 2°C to 8°C, up to 12 hours at 8°C to 25°C, protected from light. Punctured Vial: 6 hours at 2°C to 25ºC.
Vials	2 mL clear multidose vial. Each vial contains 5 doses of 0,3mL. Pack size: 195 vials.	5 ml dispersion in a vial. Each vial contains 10 doses of 0.5mL. Pack size: 10 multidose vials.
Excipients	((4-hydroxybutyl)azanediyl)bis(hexane-6,1-diyl)bis(2-hexyldecanoate) (ALC-0315), 2-[(polyethylene glycol)-2000]-N,N-ditetradecylacetamide (ALC-0159), 1,2-Distearoyl-sn-glycero-3-phosphocholine (DSPC), Cholesterol, Potassium chloride, Potassium dihydrogen phosphate, Sodium chloride, Disodium phosphate dihydrate Sucrose, water for injections.	Lipid SM-102, Cholesterol, 1,2-distearoyl-sn-glycero-3-phosphocholine (DSPC), 1,2-Dimyristoyl-rac-glycero-3-methoxypolyethylene glycol-2000 (PEG2000 DMG), Tromethamol, Tromethamol hydrochloride, Acetic acid, Sodium acetate trihydrate, Sucrose, water for injections.

However, because of the recentness of the usage of mRNA-based vaccines, possible long-term effects as well as the duration of immunity are still difficult to assess^19^. Therefore, they will play an important role in the future development of mRNA vaccines and a stringent monitoring is required in present time. Another unanswered question is whether people who have already been vaccinated and thus don’t develop the symptoms of COVID-19 can nevertheless spread the virus to other people^19^. Furthermore, studies have not yet looked enough into potential side effects of the two authorized mRNA vaccines on pregnant women^21,22^.

Many stories reporting the death of individuals after receiving vaccination have been spread over the media. Although such stories sound very deterrent and alarming, no causal link could be identified between the reported deaths and the vaccination. One example of a study conducted to investigate the death of patients shortly after receiving mRNA-based COVID-19 vaccination is the report of the Paul Ehrlich Institute in Germany, that assessed 113 reported deaths occurring between one hour and 19 days after vaccination. Firstly, it could be observed that all patients had pre-existing or previous illnesses. Thus, their death could more likely be explained by other factors than the vaccine itself. Maybe the synergy of both might have been the problem. Secondly, 20 patients died of COVID-19 disease. It could be seen that all those people except one man had only received the first dose of vaccination^28,29^. This shows the importance of completing the vaccination schedule in order to achieve protection from COVID-19 disease.

### 3.3 Production time and standardization

In contrast to some of the traditional vaccines, infectious parts of the pathogen are not part of the vaccine, but only the instruction to build such a part is. Therefore, the time needed for the multiplication, growth and inactivation or isolation of the wanted part of the virus can be saved. This traditional process also consumes a lot of material, namely the tissue the viruses are grown on (chicken or mammalian eggs)^27^. On the contrary, once the genetic sequence of the antigen is known, the mRNA-based vaccine can easily be recreated in biomedical laboratories all over the world^15,26,27^. Hence, mRNA vaccine manufacturing is not only less time-consuming but also cheaper, as laboratories are usually equipped with the required materials^27^. This also facilitates large-scale vaccine production, which is particularly important in regard to reducing the impact of pandemics^27^.

### 3.4 Storage

On the downside, mRNA-based vaccines have to be stored and transported at very low temperatures which is an obstacle for their distribution to the people. For example, Comirnaty must be kept at -90°C to -60°C, whereas Moderna vaccine can resist at slightly higher temperatures (-25°C to -15°C). These vaccines are only valid a few months (Comirnaty 6 months and Moderna vaccine 7 months). Thus, vaccine distribution has to be logistically well organized to ensure that the produced vaccines can actually reach their recipients. Once the vials have been opened, again time remaining for consumption is very little (see Table 2)^21,22^.

### 3.5 Versatility

When comparing mRNA vaccines to conventional vaccination methods (Table 3), however, a huge advantage is the possibility of modeling the mRNA and thus changing its action mechanisms inside the organism^14^.

**Table 3:** Advantages and limitations of mRNA-based vaccines and traditional vaccines

VACCINE TYPE	ADVANTAGES	DISADVANTAGES/LIMITATIONS
Existing mRNA-based vaccines	+ Efficacy: both vaccines have been shown to offer good protection from COVID-19 disease; + stronger type of immunity is provided; + safety: short in vivo half-life of the mRNA strand decreases the probability of possible side effects; + adverse effects are usually only mild; + design and production is faster and potentially cheaper since it uses less resources, although it is more expensive to distribute (special storage required); + large-scale production is possible; + mRNA strand can be adapted to fight various diseases, not only infections.	- potential side effects, especially long-term ones still have to be investigated; - efficacy against new variants is yet unknown; - still unclear, whether vaccinated individuals can infect others; - storage requires very low temperatures; - relatively short shelf life; - two vaccine shots needed.
Traditional vaccines	+ potent vaccine; + proven technology; + may not require adjuvants; + single injection often generates efficient immunity.	- manufacturing: relatively expensive, time-consuming, requires a lot of materials; - higher risk of infection during manufacturing; - difficult to scale up production.
		

Changing the molecular structure of the mRNA can yield a different rate of translation or in-vivo half-life. Purification of the mRNA further contributes to this and can also decrease immunogenicity inside the organism^14^. Another important aspect is complexing of the mRNA in different carriers to achieve a better delivery of the active agent into target cells^14^.

mRNA vaccines are highly adaptive. As mRNA is made up of the same basic building units in all organisms, a new vaccine can easily be produced by just changing the sequence of the four nucleotides to code for a pathogen-specific antigen. The only knowledge required to be able to start the process is the actual sequence coding for the antigen. This feature makes mRNA vaccines so valuable to quickly respond to outbreaks of infectious diseases, such as the actual COVID-19 pandemic^27^. Moreover, its therapeutic use in treating cancer is also being studied^27^. Here, the mRNA can code for a tumour-specific antigen and can thus teach the body’s immune system to actively fight cancer cells^27^.

## 4. Conclusion

Although COVID-19 still presents a challenge, worldwide disease prevention is making great progress thanks to the rapid development, testing and authorization of vaccines. There are many different and innovative approaches to achieve immunity, and so far, two mRNA-based vaccines have been proven to be highly effective in their clinical testing phases. Once it has reached the cell, the mRNA uses the cellular translation machinery to be expressed as the SARS-CoV-2 antigen. The body can recognize the produced protein as pathogenic and an immune response is stimulated, teaching the body’s immune cells how the critical part of SARS-CoV-2 looks like, to be able to defend the body against it. Indeed, side effects occur quite often, but in most cases, they are attributable to the generated immune response and can thus indicate that the vaccine is actually working. Nevertheless, long term effects, possible effects on pregnant women and whether the vaccines prevent their recipients from being contagious still have to be investigated and more research is required. Therefore, it is important that even people who have been vaccinated follow the rules of hygiene, maintaining a distance of 1,5 meters from others, wearing a mask in order to decrease the risk of spreading the virus.

As for the mRNA vaccines in general, they have the potential to be a very adaptive and potent instrument in reducing infections by creating immunity inside the vaccinated organisms. It is possible to modulate the aggressiveness, efficacy and other features of the vaccine by using molecular engineering techniques. Moreover, mRNA-based vaccines can be produced relatively fast, in great amounts and in a standardized fashion to fight any type of antigen the mRNA is coding for. Thus, they represent a promising tool in preventing future epidemics and pandemics and even cancer therapy. Yet, the low temperatures required for mRNA-vaccine storage remain a problem as they impede vaccine distribution. Overall, it is important to constantly re-evaluate the vaccines by carefully monitoring their consumption.

Personally, we think that the emergency authorization of the two mRNA-based vaccines is a promising step forward that can challenge and overcome the actual COVID-19 pandemic. However, there is still a lot more work to be performed.
